# Understanding Engagement in Digital Mental Health and Well-being Programs for Women in the Perinatal Period: Systematic Review Without Meta-analysis

**DOI:** 10.2196/36620

**Published:** 2022-08-09

**Authors:** Jacqueline A Davis, Jeneva L Ohan, Lisa Y Gibson, Susan L Prescott, Amy L Finlay-Jones

**Affiliations:** 1 Telethon Kids Institute Nedlands Australia; 2 School of Medicine University of Western Australia Perth Australia; 3 School of Health Sciences Curtin University Bentley Australia; 4 School of Medical and Health Sciences Edith Cowan University Joondalup Australia; 5 The Nova Institute for Health Baltimore, MD United States

**Keywords:** digital interventions, perinatal, mental health, well-being, logic model, systematic review, mobile phone

## Abstract

**Background:**

Pregnancy and the postnatal period can be a time of increased psychological distress, which can be detrimental to both the mother and the developing child. Digital interventions are cost-effective and accessible tools to support positive mental health in women during the perinatal period. Although studies report efficacy, a key concern regarding web-based interventions is the lack of engagement leading to drop out, lack of participation, or reduced potential intervention benefits.

**Objective:**

This systematic review aimed to understand the reporting and levels of engagement in studies of digital psychological mental health or well-being interventions administered during the perinatal period. Specific objectives were to understand how studies report engagement across 4 domains specified in the Connect, Attend, Participate, and Enact (CAPE) model, make recommendations on best practices to report engagement in digital mental health interventions (DMHIs), and understand levels of engagement in intervention studies in this area. To maximize the utility of this systematic review, we intended to develop practical tools for public health use: to develop a logic model to reference the theory of change, evaluate the studies using the CAPE framework, and develop a guide for future data collection to enable consistent reporting in digital interventions.

**Methods:**

This systematic review used the Cochrane Synthesis Without Meta-analysis reporting guidelines. This study aimed to identify studies reporting DMHIs delivered during the perinatal period in women with subclinical mood symptoms. A systematic database search was used to identify relevant papers using the Ovid Platform for MEDLINE, PsycINFO, EMBASE, Scopus, Web of Science, and Medical Subject Headings on Demand for all English-language articles published in the past 10 years.

**Results:**

Searches generated a database of 3473 potentially eligible studies, with a final selection of 16 (0.46%) studies grouped by study design. Participant engagement was evaluated using the CAPE framework and comparable variables were described. All studies reported at least one engagement metric. However, the measures used were inconsistent, which may have contributed to the wide-ranging results. There was insufficient reporting for enactment (ie, participants’ real-world use of intervention skills), with only 38% (6/16) of studies clearly recording longer-term practice through postintervention interviews. The logic model proposes ways of conceptualizing and reporting engagement details in DMHIs more consistently in the future.

**Conclusions:**

The perinatal period is the optimal time to intervene with strength-based digital tools to build positive mental health. Despite the growing number of studies on digital interventions, few robustly explore engagement, and there is limited evidence of long-term skill use beyond the intervention period. Our results indicate variability in the reporting of both short- and long-term participant engagement behaviors, and we recommend the adoption of standardized reporting metrics in future digital interventions.

**Trial Registration:**

PROSPERO CRD42020162283; https://www.crd.york.ac.uk/prospero/display_record.php?RecordID=162283

## Introduction

### The Importance of the Perinatal Period

Pregnancy, delivery, and the postnatal period can be times of increased psychological distress (stress, anxiety, or depression) [1], and up to 20% of women experience depression during the perinatal period [2,3]. There is considerable evidence that psychological distress during this period has detrimental effects on maternal health and can have long-term deleterious effects on the child [4-7], as recognized as part of the Developmental Origins of Health and Disease paradigm [8,9]. In particular, there is growing evidence of intergenerational transmission of poor mental health in utero and the first years of life through these pathways [10]. Therefore, cost-effective, accessible interventions that support lasting positive mental health while also preventing symptoms of mental health problems are of critical importance for public health. Mental health interventions to promote well-being have the potential to not only improve women’s outcomes but also minimize the risk of negative health effect transmission to the next generation.

Health promotion strategies aim to enable optimal health and skills to cope with adversity in well subclinical populations. Therefore, it is important that efforts are made not only to deal with illness but also to develop individuals’ emotional skills that can be applied in everyday life [11]. Psychological interventions aimed at perinatal women have also been shown to be effective when delivered digitally [12]. Digital interventions—that is, computer- or web-based interventions—can be delivered offline or on the web via a computer, tablet, or smartphone. In this format, interventions can be accessed by numerous people across wide geographical regions in a cost-effective and flexible manner [13]. Web-based interventions may be particularly useful in the perinatal period, given the accessibility issues faced by this population and as many pregnant women search the internet for health information [14,15]. Furthermore, these interventions may help overcome numerous barriers that exist for women who attempt to access traditional perinatal well-being, psychological distress prevention, or treatment programs, especially challenges in navigating psychosocial care systems [13]. Widespread restrictions imposed because of the COVID-19 pandemic have generated additional barriers to accessing mental health and well-being information and services [16].

### Digital Mental Health Interventions

Although a recent systematic review provided preliminary evidence that web-based interventions can be a promising and advisable form of intervention during the prenatal period [13], there is a paucity of evidence on the long-term effectiveness of these programs [17]. There are many issues affecting digital mental health intervention (DMHI) implementation, such as availability issues, lack of promotion by health care providers, and lack of long-term outcome data; ultimately, program engagement is key. Low uptake of effective, evidence-based programs could diminish women’s and infants’ opportunities to enhance their well-being, limiting equitable public health benefits.

Dropout from the intervention and loss to follow-up reduces the treatment effect [18]. Although it has been argued that various strategies, including email prompts, SMS text messages, and *homework* are ways of helping participants develop intervention skills that can be applied, practiced, and sustained [19], it is unclear how frequently studies of DMHIs use or evaluate these strategies. Despite studies demonstrating intervention efficacy for those that remain in the study, we argue that it is just as salient to measure engagement as a benchmark of effectiveness. Web-based interventions provide tools to learn more about participant engagement and, furthermore, how it relates to retention and intervention outcomes, both in the short and the long term. This information can be used to understand the dynamics of engagement [18] and how to strengthen these characteristics in intervention development and delivery.

### Assessing Engagement

It is widely accepted that the full benefit of many effective treatments can be achieved only if the prescribed regime is followed reasonably closely [20]. Recent reviews [21,22] have consistently highlighted these challenges with regard to low engagement and retention rates, particularly for digital programs that often experience poor reach and uptake [13]. Sustained engagement is a complex process that has been identified as a crucial factor in intervention success [23]. However, there is a lack of systematic methodologies to assess engagement, particularly in real-world contexts. Comparing program engagement across research studies is difficult because of the wide range of strategies applied to evaluate engagement outcomes [22,24]. Accordingly, applying structured processes to assess engagement can make comparisons more meaningful.

One of the frameworks for evaluating engagement in face-to-face programs, which can be adapted to web-based programs, is the Connect, Attend, Participate, and Enact (CAPE) model [25]. The CAPE model identifies and defines 4 aspects of engagement at various stages of intervention. First, *connect* pertains to how many people express interest in engaging in an intervention out of those eligible. Second, *attend* refers to continuous presence, such as the number of intervention sessions a participant completes. Third, *participat*e is the degree to which participants actively engage with the content of the intervention, such as completing intervention tasks and remaining in the program. The final component, *enact*, refers to the participant making use of intervention strategies or knowledge as part of their daily life. Although this was developed to guide face-to-face parenting program engagement research, it can be readily applied to understanding and measuring digital intervention engagement in a research context.

### Objectives

At a time when public resources are strained, policy makers and program administrators are looking to invest in effective, engaging prevention programs supported by scientific evidence and delivering long-term benefits. Intervention engagement must be foremost among these considerations, as this will ultimately determine the degree to which the target population takes up and benefits from the intervention when implemented in the community. Systematic reviews are an influential decision-making tool as they summarize a body of scientific research; identify implications for policy and practice [26,27]; and can be used to guide investment decisions, particularly for complex problems, such as poor intergenerational mental health.

This systematic review aimed to understand the reporting and levels of engagement in studies of web-based psychological mental health or well-being interventions administered in the perinatal period to women with subclinical mood symptoms.

Specifically, we aimed to (1) understand how studies report engagement, with engagement defined as containing the 4 steps in the CAPE model; (2) make recommendations on best practices to report engagement in DMHIs based on this; and (3) understand levels of engagement in intervention studies in this area.

To maximize the utility of this systematic review, we intended to develop practical tools for future public health use: to develop a logic model from the literature to reference the theory of change, evaluate the studies using the CAPE framework, and develop a guide for future data collection to enable consistent engagement reporting in web-based (and offline) interventions.

## Methods

The methods used in this systematic review combine standard rigorous and transparent review methods using the Cochrane Synthesis Without Meta-analysis (SWiM) reporting guidelines [28] in conjunction with the development of a logic model to understand the theory of change.

### Search Strategy

The review question, search strategy, inclusion criteria, and methods were registered in PROSPERO (International Prospective Register of Systematic Reviews; approval number CRD42020162283). The research question was as follows: what is known about engagement in digital mental health and well-being programs for women in the perinatal period? A systematic database search was conducted to identify papers relevant to the aims of this review. The initial search was performed by the first reviewer (JAD), using the Ovid Platform for MEDLINE, PsycINFO, EMBASE on the EBSCO Platform, Scopus, Web of Science, and Medical Subject Headings on Demand for all English-language articles published in the past 10 years (ie, from January 1, 2010, to May 29, 2020). Keywords and index terms identified as relevant in the search strategy were used and individual search criteria were developed for each database. All the database search strategies are provided in Multimedia Appendix 1. The impact of the COVID-19 pandemic delayed this publication; therefore, a subsequent rapid review was undertaken in May 2022 and performed in Google Scholar Advanced search to elicit any further publications since June 2020.

### Identification of Studies and Eligibility Criteria

The search strategy aimed to identify studies reporting on engagement and retention in digital mental health and well-being programs for women during and after pregnancy. Clear inclusion and exclusion criteria were developed using the Population, Intervention, Comparison, Outcomes, and Study framework to guide the inclusion criteria for participants, intervention or phenomena of interest, comparators, outcomes, study design, and context (Textbox 1).

**Inclusion and exclusion criteria (based on the Population, Intervention, Comparison, Outcomes, and Study framework).**

**Inclusion criteria**

- Participants
  - Childbearing individuals in the perinatal period (ie, from conception to the first year of the infant’s life)
  - Studies focusing predominantly on the childbearing individual but can include partners
  - Studies that include childbearing individuals at moderate risk for psychological distress (ie, with Edinburgh Postnatal Depression Scale score ≤12)
  - Studies that include women at risk of postnatal depression with a history of depression or anxiety (ie, early intervention)
- Intervention
  - Any minimal contact digital interventions provided in the perinatal period aiming to reduce mild to moderate psychological distress or promote psychological well-being (ie, minimal contact as defined by a maximum of <1 hour of direct contact each week)
- Comparators
  - Studies with any form of comparator were considered
- Outcomes
  - None; although the focus of the review was on engagement outcomes, we included any studies of interventions meeting the above criteria to determine the proportion that reported engagement outcomes
- Study design
  - Quantitative (eg, randomized controlled trials, quasi-experimental studies, cohort studies, descriptive studies), and qualitative studies

**Exclusion criteria**

- Participants
  - Studies considering programs before conception and those specifically targeting the child
  - Studies focusing predominantly on the partner or father
  - Studies that include women at high risk for psychological distress (ie, with Edinburgh Postnatal Depression Scale score ≥13)
- Intervention
  - Interventions with a primary focus other than mental health or well-being (eg, parenting self-efficacy)
  - Interventions delivered face to face or as telehealth or telephone coaching

### Selected Studies

All papers that appeared eligible based on their title and abstract were retrieved for screening. The first author (JAD) reviewed the titles and abstracts of all papers, assessed eligibility, and noted any reasons for exclusion. Full-text articles were assessed for eligibility and reviewed independently by both the first author (JAD) and third author (LYG). Once the third author (LYG) had reviewed the papers, any discrepancies were resolved through team discussion. The reference lists of the included studies were examined to identify additional relevant papers.

### Coding of Study Characteristics and Data Extraction

Key article characteristics were recorded using a Microsoft Excel (version 2020) data extraction table developed for this review. These characteristics included general information about the study, such as the country and author, along with specific information about the study design, comparators, and intervention type. Coding of the study characteristics enabled us to group the studies as part of our synthesis. As our primary aim was to understand the engagement of the study population, we characterized the assessment time points, engagement measures, and reporting of attrition and adherence. Data relevant to engagement were extracted using the CAPE framework; this included variables for recruitment, retention, attrition, and follow-up time points. A framework analysis methodology [29] was used to determine which variables should be included in each step of the CAPE framework.

### Development of Logic Model

**Figure 1:**
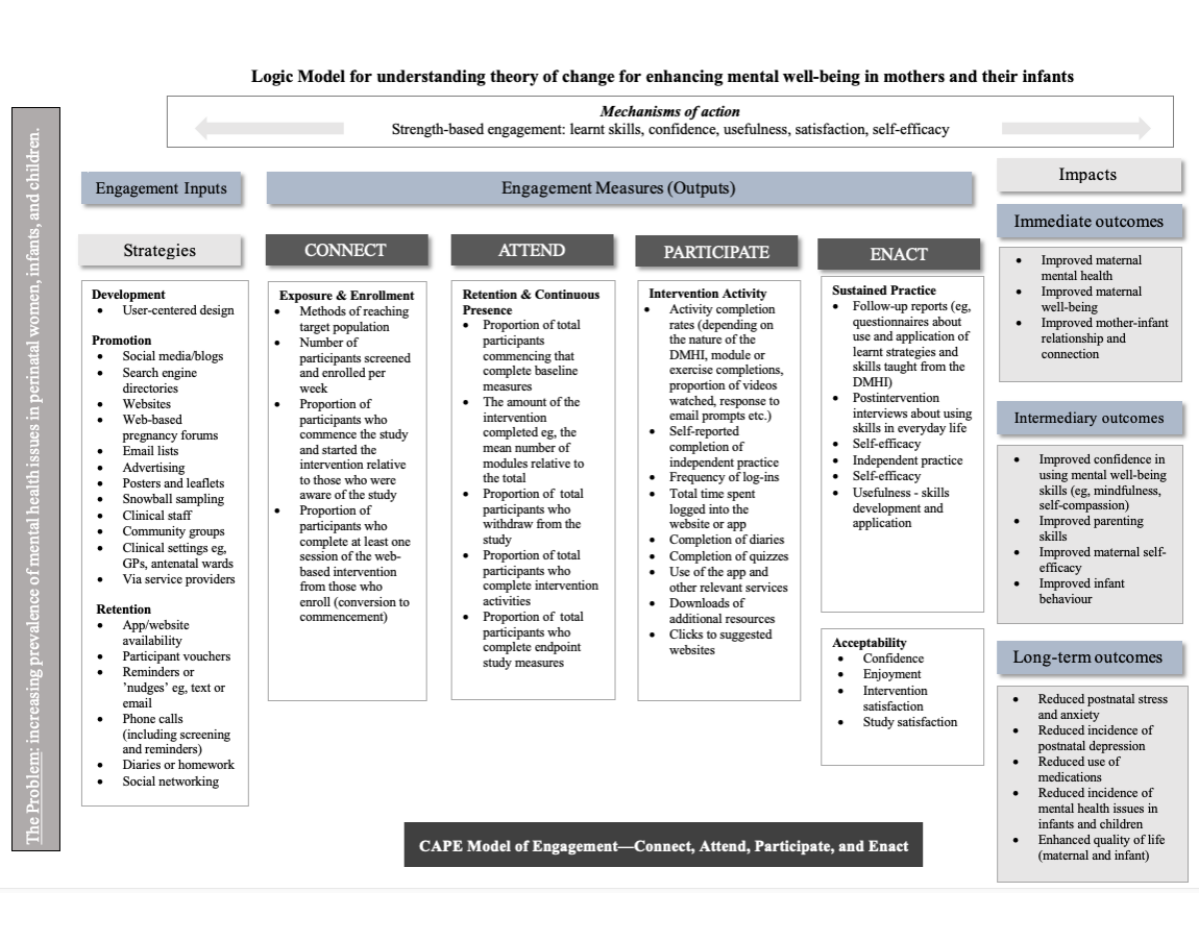
Proposed logic model. GP: general practitioner; DMHI: digital mental health intervention.

Logic models can help conceptualize a complex review question and specify analytic links to test the plausibility that a program works as intended [26]. Logic models typically illustrate the chain of reasoning underpinning how interventions lead to immediate (or short-term) outcomes and then to longer-term outcomes and impacts [30]. A key part of the model is detailing the mechanisms of change within the pathway and the moderating and mediating factors that may be associated with or influence outcomes. This is often referred to as the theory of change [30]. In this systematic review, the research team developed a logic model to aid the process of understanding how and when the CAPE framework could be applied to interpret the role of different engagement variables in promoting outcomes in digital perinatal mental health and well-being programs. The project team collaboratively developed the logic model, drawing on themes in the literature and the team’s collective knowledge and experience. To develop the logic model, we incorporated the types of engagement metrics found in the selected studies that could be used to assess engagement (Figure 1).

### Quality Appraisal and Risk of Bias

The risk of bias for studies included in this review was based on the Cochrane Collaboration’s tool for assessing the risk of bias for each category of study (ie, randomized controlled trials [RCTs] and non-RCTs), and the risk of bias was adapted for this review and classified as low, uncertain, or high based on the Cochrane risk of bias tool [31] and the primary aim of this systematic review (ie, engagement rather than efficacy). The assessment of study quality was undertaken by the first author (JAD) and reviewed by the project team. Multimedia Appendix 2 [17,32-46] provides the detailed risk of bias assessments of the included studies.

### Synthesis of Results

As this systematic review synthesized the results from a diverse range of interventions, we used SWiM guidelines [28] to promote transparent reporting. The SWiM items enable studies to be grouped and provide guidance on the reporting of standardized metrics used for the synthesis of findings. Specifically, we undertook the following steps:

1. Summarized the characteristics of each study and reported intervention implementation, recruitment and engagement activities, study findings, reported attrition, and methodological quality
2. Determined which studies were similar enough to be grouped within each comparison by comparing across studies (eg, types of digital platform and postnatal vs antenatal)
3. Determined which data were available for synthesis
4. Synthesized the characteristics of the studies
5. Performed a statistical synthesis for appropriate quantitative data and comprehensive critical appraisal through a meta-synthesis approach for qualitative data
6. For each trial included in this systematic review, we recorded counts of trial participants who were assessed for eligibility, those who were recruited, and those who were allocated to the intervention and control arms; rates of recruitment, trial completion, and loss to follow-up were synthesized by evaluating the proportion of recruitment, completeness, and loss to follow-up in base R (R Foundation for Statistical Computing) statistical package [47]; synthesized data were reported as forest plots [48].

## Results

### Included Studies

The electronic searches generated a database of 3473 potentially eligible studies that were assessed using the review eligibility criteria. After duplicates were removed (680/3473, 19.58%), all titles and abstracts were screened for eligibility. In total, of the 3473 studies, 2795 (80.48%) records were screened, and 2654 (76.42%) were excluded based on the inclusion or exclusion criteria (Textbox 1). After the first screening, 5.31% (141/2654) of potential studies remained; the full-text articles were assessed for eligibility by the first and third authors. Of the remaining 141 studies, 125 (88.7%) were excluded on consensus by the project team; the first and third authors independently screened the papers that were verified by the team, resulting in a final selection of 16 (11.3%) studies to be included in the synthesis. The final studies were then grouped according to the study design.

**Figure 2:**
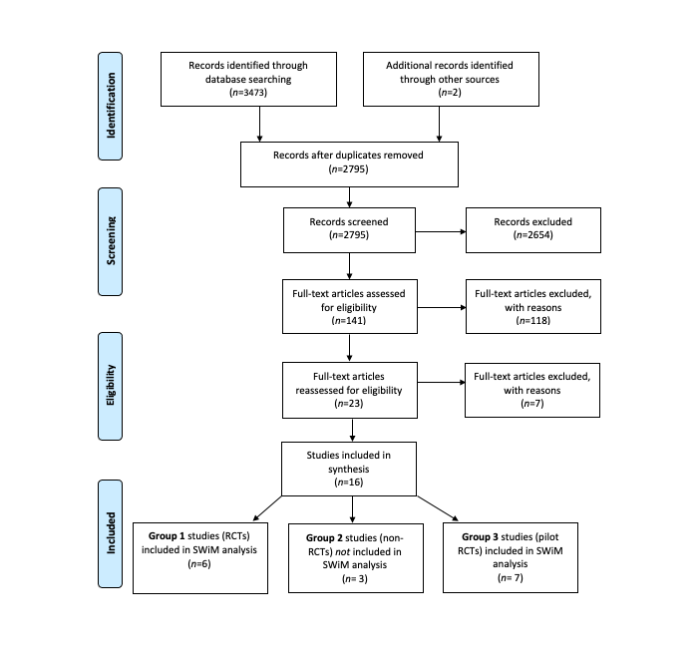
Search flow diagram (PRISMA [Preferred Reporting Items for Systematic Reviews and Meta-Analyses]). RCT: randomized controlled trial; SWiM: Synthesis Without Meta-analysis.

The literature search and inclusion processes are detailed in the PRISMA (Preferred Reporting Items for Systematic Reviews and Meta-Analyses) flow diagram (Figure 2). The search flow diagram indicates the papers that were selected for synthesis using the PRISMA guidelines [49]. A secondary rapid search conducted in 2022 did not yield any additional papers that met our specific inclusion criteria.

### Synthesis of Results

#### Overview

The primary aim of this systematic review was to assess the engagement of women participating in digital mental health or well-being interventions; therefore, the standardized metric and transformation method [28] used across all studies were those used to evaluate engagement rather than well-being effect sizes. On analysis of study characteristics, we grouped the 16 interventions according to study design to provide a cohesive comparison in a broad range of study types. Group 1 was RCTs (6/16, 38%), with active and control arms. Generally, RCTs had a longer follow-up period (maximum of 12 months). Group 2 included non-RCTs (3/16, 19%), with no active comparators and brief or no follow-up period. Group 3 comprised pilot RCTs (7/16, 44%) with active and control arms and a follow-up period.

#### Study Characteristics

In all groups, there was a range of therapeutic approaches, including cognitive behavioral therapy, parenting education, positive psychology, mindfulness, and compassion-based training. Several studies used a psychoeducation approach to build parenting self-efficacy, such as Chan et al [50], Shorey et al [51], Corno et al [32], and Tsai et al [34]. In group 1, overall, 25% (4/16) were delivered as self-help internet interventions and 13% (2/16) as smartphone-based mobile apps; in group 2, all were delivered as self-help internet interventions; in group 3, all were delivered as internet interventions apart from the study by Barrera et al [37], which was delivered as an SMS text messaging program. In addition, 13% (2/16) of studies included the assessment of physiological biomarkers: Cornsweet [38] and Matvienko-Sikar and Dockray [41]. A summary of the study characteristics is reported in Tables 1-3 (a more detailed report is available in Multimedia Appendix 2, including intervention outcomes). For this review, we reported engagement measures collected for each study as CAPE metrics.

**Table 1:** Group 1: randomized controlled trials (N=6).

Intervention type, format, and duration	Study aims (sample size)	Engagement measures: connect	Engagement measures: attend	Engagement measures: participate	Engagement measures: enact
Self-guided; iWaWa^a^; 9 modules [13]	Assess the feasibility and acceptability of iWaWA among postpartum women with anxiety (89 participants)	Assessed for eligibility (n=147): recruited via social media, posters, and flyers and numbers recruited Reasons for exclusion 89 enrolled and randomized to treatment and control	Engagement with internet-based components Attrition and attendance Participant CONSORT^b^ flow diagram (access, allocation, and follow-up)	Module views, module completion, number and duration of support calls	Treatment feasibility (engagement and usability) and acceptability (usefulness, satisfaction, and helpfulness) were assessed after treatment through semistructured interviews
Smartphone-based mobile app [50]	Assess the difference in the levels of antenatal and postnatal depression in participants (660 participants)	Assessed for eligibility (n=803) Reasons for exclusion 660 enrolled and randomized (intervention or treatment as usual)	Participant CONSORT flow diagram (eligibility, enrollment, randomization, follow-up, and analysis) Retention rates	The use of the app and other relevant services (eg, antenatal classes and other pregnancy resources: books and websites) documented by self-report	Postintervention survey included Use of the app
Web-based compassion-based intervention; *Kindness for Mums Online*; 5-6 weeks [52]	Assess the effect of the intervention on participants’ well-being (206 participants)	Assessed for eligibility (n=310) Recruitment methods: social media and snowball sampling Participant vouchers Accessibility Reasons for exclusion 206 enrolled and randomized	Participant CONSORT flow diagram (enrollment, allocation, follow-up, and analysis)	Reporting of attrition and engagement (ie, completion of sessions and frequency or program use)	Acceptability: participants were asked to rate the ease of use and satisfaction after the intervention
A Chinese version of the MBSP^c^ program; 10 hours of training with 36 episodes; 6-week internet-based intervention [53]	Assess the effect of the mindful self-compassion intervention on preventing postpartum depression in a group of symptomatic pregnant women (314 participants)	Assessed for eligibility (n=472) Screening and baseline assessment (n=344) Reasons for exclusion Randomized (n=314)	Participant CONSORT flow diagram (eligibility, allocation, follow-up, and analysis) Attendance rates Reporting of retention	Reporting of attrition Feasibility and acceptability After completing each exercise, participants were instructed to exercise the steps during the day; participants provided a graphical overview and a web-based diary book where they registered their reflections	Reporting of retention and attrition after the intervention
Condensed web-based version of an 8-week mindfulness course; “Be Mindful Online”; 4 weeks on the web [54]	Evaluate the potential of a web-based mindfulness course for expectant participant women (185 participants)	Assessed for eligibility (n=237) Recruitment methods (email lists, social media advertising, and posters in community settings) Reasons for exclusion Enrolled and randomization methods	Participant CONSORT flow diagram (recruitment, allocation, follow-up, and analysis)	Regular reminders to log on or contact the research team via email Reporting of retention and attrition	Postcourse evaluation 45 days after baseline
Mobile app for psychoeducation and postnatal depression; “Home-but not Alone” [51]	Examine the effectiveness of the program in improving participant parenting outcomes (250 participants [couples])	Assessed for eligibility (n=360 couples) Reasons for exclusion Recruitment methods Randomization methods to intervention or control	Participant CONSORT flow diagram (eligibility, recruitment, allocation, follow-up, and analysis)	The research team monitored the use of the app and parents received reminders each week	Intervention posttest

^a^iWaWa: internet-based What Am I Worried About.

^b^CONSORT: Consolidated Standards of Reporting Trials.

^c^MBSP: Mindfulness-Based Strengths Practice.

**Table 2:** Group 2: non–randomized controlled trials—case series, open trial, and quasi-experimental (N=3).

Intervention type, format, and duration	Study aims (sample size)	Engagement measures: connect	Engagement measures: attend	Engagement measures: participate	Engagement measures: enact
Positive psychology web-based intervention; 5-week web-based self-applied positive psychology intervention specifically adapted for pregnant women; 4 modules [32]	Examine the effect of a positive psychology web-based intervention on indices of participants’ prenatal well-being (6 participants); case series design	Eligibility and recruitment method Preassessment on the web	Weekly emails—reminders for assessments	Compliance with the intervention measure was developed by the research team No reported attrition	Exercise preferences were assessed at the posttest time point
Internet program plus weekly phone coaching sessions, individually or group-wise; MMB^a^ program; 8 weeks [33]	Examine the feasibility, acceptability, and preliminary outcomes of MMB for use with pregnant women at risk for depressive relapse (37 participants); open trial—no control group	Assessed for eligibility (n=48) Reasons for exclusion Recruitment methods—flyers and via service providers Prescreening by phone Intake interview in person or by phone Participant enrollment and flow (eg, reasons for declining to participate)	Participant CONSORT^b^ flow diagram (eligibility, enrollment, follow-up, and analysis)	Session completion and participation in phone coaching calls Home practice completion Participant engagement (eg, completion of sessions, practice per week, and time)	Self-reported satisfaction (perceived benefits and challenges) via questionnaire and engagement interview (qualitative) at session completion
Web-based modules: web-based maternity health records, antenatal health education, self-management journals, and infant birth records [34]	Investigate the effectiveness of a web-based antenatal care and education system on pregnancy-related stress, general self-efficacy, and satisfaction with antenatal care (135 participants) quasi-experimental design	Eligibility—control (n=75) and experimental (n=80) group at pretest Recruitment methods (convenience sampling) Assignment methods to experimental or control groups	Participant CONSORT flow diagram (enrollment, follow-up, and analysis) Attrition	Assistance was offered via telephone, email, web conferencing, or face-to-face guidance Follow-up phone calls were made to the participants Attrition	N/A^c^

^a^MMB: Mindful Mood Balance.

^b^CONSORT: Consolidated Standards of Reporting Trials.

^c^N/A: not applicable.

**Table 3:** Group 3: pilot studies (N=7).

Intervention type, format, and duration	Study aims (sample size)	Engagement measures: connect	Engagement measures: attend	Engagement measures: participate	Engagement measures: enact
Brief web-based self-help intervention—5 components considered effective in challenging negative beliefs [35]	Assess positive mood in participating mothers of babies and toddlers (80 participants)	Eligibility Recruitment methods—internet, leaflets, and community postnatal groups Randomization methods	Only 1 session	Compliance (missing data)	Acceptability—an open-response question at the end of the intervention (qualitative) Implications for policy and practice
Automated self-help internet intervention; 8 lessons—accessible anytime [36]	Assess the efficacy of the intervention to reduce the risk of postnatal depression in participating women (111 participants)	Assessed for eligibility (n=5071) Consented (n=2966) Recruitment methods—web-based search engine directories, (eg, Google advertisements “sponsored links”) Randomization methods Initial log-ins to the website	Participant CONSORT^a^ flow diagram (eligibility, consent, allocation, follow-up, and analysis) Adherence	Automated email messages Automated self-help via website Log-ins, total time spent logged into the website, and the last lesson viewed recorded Module feedback on the materials viewed (eg, usefulness and understandability) Attrition	Includes discussion on experience and engagement and feedback assessment
Minimal contact automated SMS text messaging; *BabyText* program [37]	Assess acceptability of an SMS text messaging program to prevent postpartum depression (10 participants [pregnant and postpartum women])	Eligibility Recruitment methods—flyers at general public bulletin boards and community agencies; websites and blogs	Compliance	Attrition	Feedback assessment (qualitative) Acceptability assessment
Intervention—self-guided; 15 steps, each of which takes 45 minutes [38]	Assess feasibility and acceptability; study 1 (n=6): effects of a single teaching and biofeedback session on maternal and fetal biofeedback; study 2 (n=9): effect of consumer satisfaction	Study 1: eligibility and recruitment methods (flyers at antenatal classes) Study 2: eligibility and recruitment methods (flyers at antenatal classes)	Study 1: compliance with baseline and 2 conditions (teaching and practice) Study 2: compliance to complete 15 steps	Attrition Feasibility and acceptability	Study 1: no postintervention measures Study 2: postintervention assessment and interview Qualitative follow-up
8-week web-based prevention intervention; website plus initial phone call; 16 core didactic lessons plus 3 postpartum booster sessions and 5 associated tools [39]	Assess a CBT^b^ peer support intervention to prevent postnatal depression in participants (24 participants)	User-centered-design, recruited via flyers Assessed for eligibility (n=216) Completed baseline assessment (n=30) Enrolled and randomization methods	Participant CONSORT flow diagram (screened, completed the baseline assessment, and enrolled) Adherence	Email notifications Total log-ins and completion of tools and lessons Peer support features (likes, comments, nudges, and posts) Reporting of attrition and site use (log-ins); usability and acceptability	Usability and satisfaction (Usability, Satisfaction, and Ease of Use questionnaire)
Self-guided, web-based intervention to prevent postpartum depression symptoms; *Be a Mom*; 5 modules [40]	Explore the processes underlying therapeutic change for participants in the intervention (194 participants)	Assessed for eligibility (n=643) Email invitation to participate Recruitment methods—in person and web-based Reasons for exclusion Baseline assessment (n=241) Randomization methods (intervention or waitlist control)	Participant CONSORT flow diagram (eligibility, enrolled, randomized, and follow-up) Adherence	Email reminders after 7 days without accessing intervention Attrition	Postintervention measures included emotion regulation, psychological flexibility, and self-compassion
Web-based mindfulness and gratitude intervention 4 times a week for 3 weeks [41]	Assess the effect of a novel gratitude and mindfulness-based intervention on prenatal stress, cortisol levels, and well-being in participating women (46 participants)	Assessed for eligibility (n=362) Recruitment methods—posters, leaflets, and pregnancy forums Reasons for exclusion Randomization methods SMS text message reminders No additional contact with the study team during the study period	Participant CONSORT flow diagram (enrollment, allocation, follow-up, analysis)	Participant adherence was evaluated as the total frequency of completion of the web-based diary entries Proxy measure for full intervention use	Limitations in fidelity evaluation

^a^CONSORT: Consolidated Standards of Reporting Trials.

^b^CBT: cognitive behavioral therapy.

### Variables Assessed to Evaluate Engagement

#### Overview

Participant engagement was evaluated using the CAPE model of engagement [25], which is described in more detail in the following sections. Three-quarters of all the studies included a CONSORT (Consolidated Standards of Reporting Trials) participant flow diagram with similarities in reporting (ie, enrollment, allocation, follow-up, and analysis). In terms of strategies to prompt engagement and promote retention (attendance and participation), various methods were used and reported, including email and text reminders, peer support features, and phone calls to participants.

#### Connect: Exposure and Enrollment

*Connect* was operationally defined as the proportion of participants who entered the study and started the intervention relative to those who were aware of the study. Although many studies reported the *exposure* methods for the target audience, for example, advertising via Facebook, Twitter, web-based email lists, community sites (medical and retail), and third-party websites (Ashford et al [13], Krusche et al [54], and Felder et al [33]), there were rarely reporting of the total population size exposed to advertising and other recruitment means. Many studies reported eligible participants who made the initial contact. For example, Barrera et al [36] reported eligible participants (n=5071) as female, pregnant, aged ≥18 years, and interested in the study website for personal use. From this total group, 2966 participants went on to participate by signing an informed consent form. A further 2114 potential participants were excluded, and the reported reasons included website error, current or missing status of major depressive episodes, and incomplete baseline. The final number of randomized participants was 852. Given the substantial drop-off between exposure to recruitment methods and randomization, reporting metrics at each stage of this process can highlight where efforts must be targeted to increase engagement.

All studies in this review reported enrollment rates in the intervention, which we defined as those who commenced the intervention relative to those who expressed interest in the study. Conversion to commencement was based on multiple factors, not just the participants’ decision to engage, both dependent and independent of the inclusion or exclusion criteria. Most studies in this review reported reasons for exclusion, ranging from lack of contact or completion of baseline surveys to elevated mental distress scores. Enrollment rates varied from a high rate of 82% (Chan et al [50]; group 1) commencing from the eligible study sample, with the lowest enrollment rate at 12% (Duffecy et al [39]; group 3). Generally, there were higher enrollment rates in the group 1 studies (clustering approximately 60%) than in group 3 (clustering approximately 25%). Only one of the studies reported strategies designed to increase enrollment. Duffecy et al [39] undertook a user-centered design process before the pilot trial to engage women from the target population in the intervention-building process. However, this study also reported the lowest enrollment rate.

#### Attend: Retention and Continuous Presence

In face-to-face interventions, *attendance* refers to the proportion of the sessions attended by each participant. Ideally, for digital interventions, attendance would be a measure of the amount of intervention completed (eg, the mean number of intervention modules relative to the total number of modules) or similar, such as the number of participants who completed all web-based interventions. This information was not stated in any of the studies included in this review. As a result, we calculated a proxy for intervention *attendance* as study attendance, operationally defined as intervention retention and continuous presence (continued interaction with the intervention), in both the intervention and control arms (where controls were used). All studies reported retention in terms of the rate of those who enrolled versus those who completed the study.

**Figure 3:**
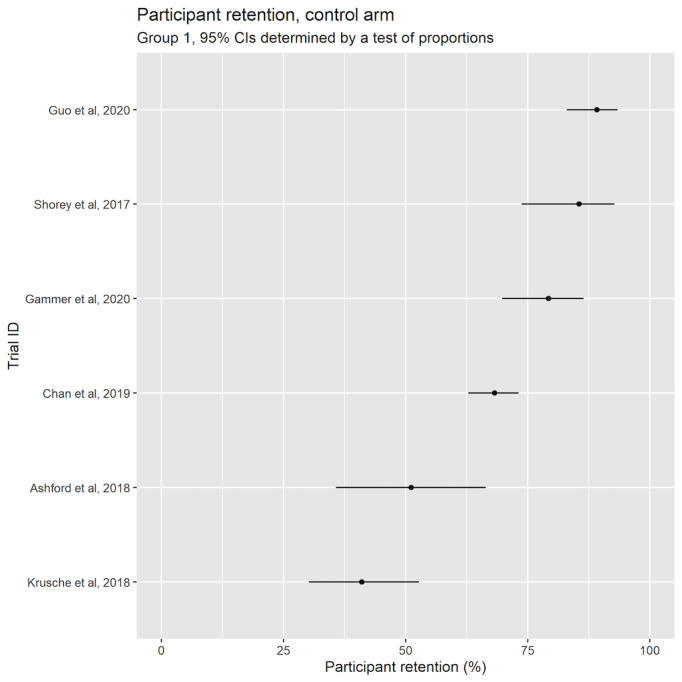
Participant retention in the intervention arm (group 1); 95% CIs determined by test of proportions [13,50-54].

**Figure 4:**
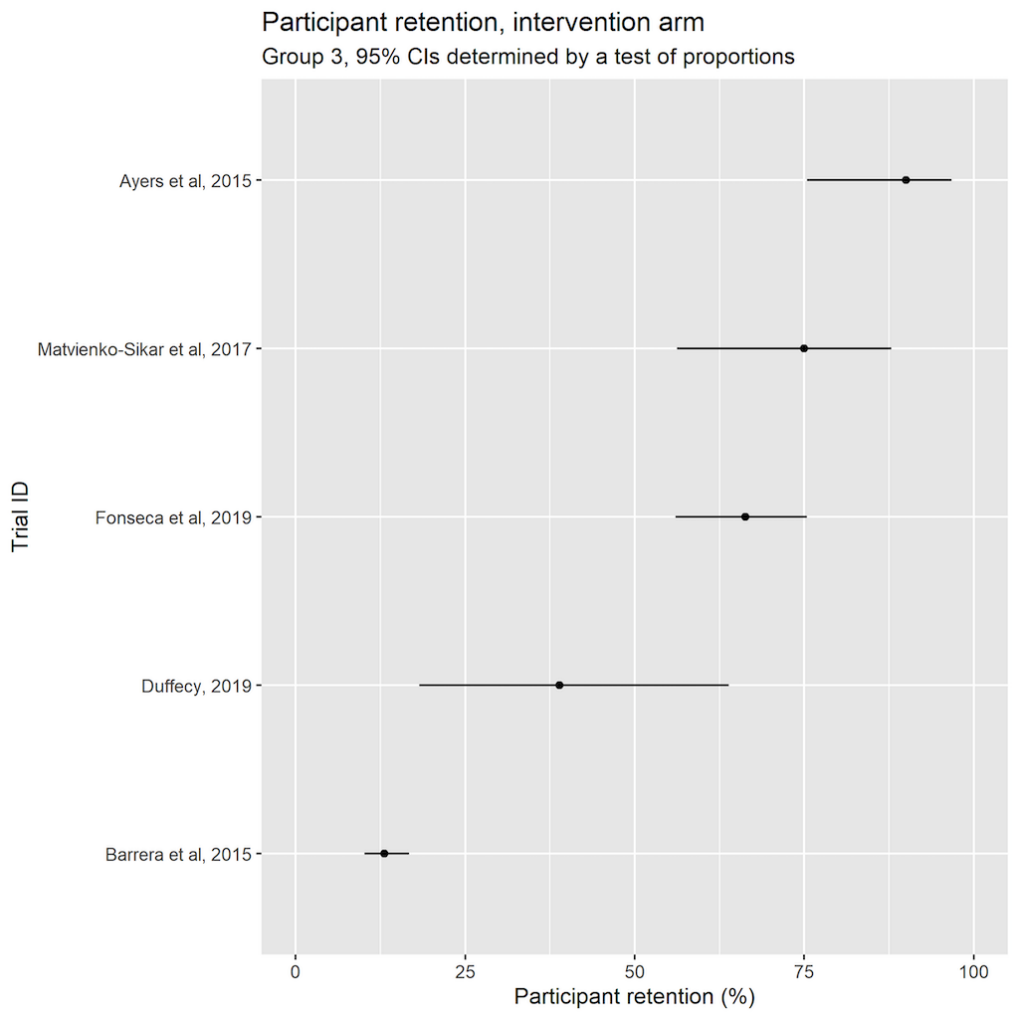
Participant retention in the intervention arm (group 3); 95% CIs determined by test of proportions [35,36,39-41].

The highest reported study retention (groups 1 and 3) was reported by Ayers et al [35] at 90%. Barrera et al [36] had the lowest intervention retention at 13%. (Figure 3 [13,50-54] and Figure 4 [35,36,39-41]).

In the control arm, Guo et al [53] had the highest participant retention rate of 89%, whereas Barrera et al [36] had the lowest at 13%.

#### Participate: Active Engagement

*Participation* was operationally defined as the completion of intervention activities; that is, *active engagement* with the intervention material. Follow-up prompts to encourage participation varied across studies; however, there were mostly weekly reminders such as SMS text messages, emails, and phone calls. A broad spectrum of metrics was used to report ongoing participation in each study, including module views, module and home practice completions, engagement with web-based components, use of the app (frequency of log-ins) and other relevant services (eg, antenatal classes), number and duration of support calls, and web-based diary entries. The heterogeneous nature of recording these activities is appropriate for the types of intervention strategies but limits our ability to consistently report and compare across studies.

#### Enact: Sustained Practice (Leading to Long-term Well-being Effects)

The limited follow-up period restricted our ability to report against measures indicating that participants applied and practiced learning skills [24]. Immediate postintervention follow-up was reported by all studies through a combination of self-report assessments, interviews, program accessibility, acceptability, and satisfaction; however, this did not necessarily include commentary on putting skills into practice. Approximately 31% (5/16) of studies undertook postintervention interviews to unpack outcomes such as usefulness, satisfaction, experience, and engagement. These interviews contributed more robust information to the user experience and provided some insight into the reasons for participation or enactment (or lack thereof) but not necessarily on the enactment itself (ie, use of the intervention skills). For example, internet-based What Am I Worried About (Ashford, 2018) was experienced as not user-friendly enough, too long, and not smartphone-friendly. Parts of the content were experienced as not always relevant or appropriate. The participants felt that the program could be improved by having it in a smartphone app format and by making the content more concise and inclusive of different parenting styles. Guo et al [53] had the highest participation rate and longest follow-up period (12 months post partum), and participants rated the program as highly useful.

### Logic Model Development

Through the analysis and reporting of each study, we recorded the types of quantitative and qualitative measures found in the selected studies that could be used to measure engagement. This enabled us to systematically construct a logic model based on our understanding of how interventions are expected to work. This was particularly pertinent for this systematic review as we did not perform a meta-analysis. As indicated, we grouped variables related to the CAPE framework; the logic model includes a range of metrics that could be systematically reported when synthesizing engagement data to visually interpret the underlying theory of change.

The logic model (Figure 1) contains 6 columns detailing the intended pathway from inputs (engagement strategies) to long-term outcomes or impacts. This approach takes a long-standing view of interventions to achieve their intended consequences. To build this model, we incorporated the types of measures undertaken in each study in this systematic review, as well as a broader range of CAPE measures found in the wider literature. The logic model indicates the point in the pathway at which the data should be collected. Mechanisms of action are factors that may facilitate engagement using a strength-based approach.

As part of this systematic review, we aimed to develop a guide for future data collection to enable consistent engagement reporting in web-based (and offline) interventions. Table 4 outlines a range of metrics that could be consistently applied in future data collection and reporting to enhance understanding of engagement and enable comparative intervention assessments.

**Table 4:** Proposed reportable metrics: engagement.

CAPE^a^ model of engagement	Measures	Definitions
Connect	Exposure and enrollment (rates should be reported for each trial arm separately)	Defined target population (ideally with population size if available) Methods of recruitment and size or proportion of the population exposed to each recruitment method Enrollment rate: proportion of participants who start the intervention relative to those who are exposed to the intervention and those who provide consent for the study Connection rate: proportion of recruited participants electing to enroll relative to those who are eligible
Attend	Intervention retention	Proportion of participants who complete the intervention relative to those who enroll in the intervention Mean, SD, and range of the number of modules completed
Participate	Intervention activity	Active engagement (depending on the nature of the intervention; this may be module completions, exercise completions, proportion of videos watched, and response to emails) Log-ins (frequency and duration) Time spent logged into the website or app Use of recommended resources (eg, downloads of additional resources and clicks to suggested websites)
Enact	Sustained practice	Follow-up reports (eg, questionnaires about the use and application of learned strategies or skills taught from the DMHI^b^) Postintervention interviews about using skills in everyday life Sustained behavior change

^a^CAPE: Connect, Attend, Participate, and Enact.

^b^DMHI: digital mental health intervention.

## Discussion

### Principal Findings

In this systematic review, we categorized the selected studies according to study type and assessed their individual and pooled characteristics. We applied the CAPE framework [25] to all studies so that we could collectively assess and compare *connection, attendance, participation,* and *enactment*. Reporting of connection and attendance measures was fairly standardized across studies and frequently reported using a CONSORT diagram; therefore, the number of people who were eligible and expressed interest in participating, proportion of participants recruited and entered in the study, and proportion of participants who were randomized and followed up, including *treatment not started* and attrition rates, was clear. Approximately 75% (12/16) of studies, provided a CONSORT participant flow diagram (indicating aspects of attendance); however, the reporting categories and terminology varied between diagrams. In general, the least frequently reported domain was enactment (ie, real-world use of intervention skills), with only 38% (6/16) of studies clearly recording and reporting results such as satisfaction, usefulness, helpfulness, and perceived benefits of the skills learned in the intervention.

Some studies reported strategies to increase *connection*. For example, Ashford et al [13] included specific details on recruitment methods, such as social media platforms, parenthood websites, and the use of posters and flyers in clinical settings. However, other studies reported minimal details of recruitment methods (eg, Guo et al [53]). No study reported information on the background target population size; that is, the total potential pool of eligible participants. This might be a useful future metric to determine and report as an indicator of the total target population reach and the effective strategies that engage them.

As previously defined, *attendance* is a measure of DMHI completion through modules or similar exercises. As this was not definitively reported, for *attendance*, we calculated a proxy for intervention *attendance* as study attendance, operationally defined as intervention retention and continuous presence, both in the intervention and control arms (RCTs). Guo et al [53] had the highest participant retention and the lowest lost to follow-up participant rate in both the intervention and control groups; the intervention group showed significant improvement in depressive and anxiety behaviors. The women in this study were at a higher risk of presenting with psychological distress at baseline; although they fit our inclusion criteria, they may have had increased motivation to attend. Duffecy et al [39] undertook a user-centered design process before the pilot trial to engage women from the target population in the intervention-building process and consult on aspects such as topics, sites, and usability of potential applications. In theory, this should support *attendance* by reducing attrition and improving retention; however, dropout from baseline assessments to 6 weeks post partum was high (63%) [39]. We recommend that future studies report both *intervention* attendance and *study* attendance as they are distinct metrics.

A key concern in web-based interventions is the lack of *participation* [18]. Logs of access and use of web-based interventions can give researchers insight into people’s behavior. As Piotrowska et al [25] suggest, “The CAPE model proposes that despite the immense importance of connecting with parents and encouraging their attendance, it is active participation that has the greatest impact on parenting.” Digital interventions provide tools for learning more about participant engagement and how this relates to retention and intervention outcomes, as well as how they might be improved through the use of different ongoing engagement strategies. Crouper et al [18] quantified participant engagement using data such as dosage, exposure, or adherence. In this systematic review, few studies reported clear metrics for participation, with the exceptions of Duffecy et al [39] and Barrera et al [36]. Other suggested metrics for future research include downloading suggested resources or websites, watching suggested videos, completing quizzes and homework, or other metrics that indicate that the participant is continuously engaging with the intervention. Additional features that have potential but continue to be underexplored and underused include chatbots, games, storytelling, rewards, avatars, and personalization [42]. These features could be developed to improve participation in interventions, general app use, and studies.

*Enactment* is difficult to define but should be represented by measures indicating that participants put what they learned from the program into practice [24]. The limited follow-up of these studies restricted our ability to report these criteria. Only one study, Guo et al [53], followed up for any length of time, and only 31% (5/16) of studies conducted exit or follow-up interviews [13,33,35-37]. These interviews contributed more robust information on the user experience. Studies assessing skill development and use underscore the potential pathways in self-guided internet therapy, such as cognitive behavioral therapy, as mechanisms of positive clinical change [43,44]. Although these studies target clinical groups, they contribute to reinforcing the need to capture behavioral skill adaptations beyond the duration of the intervention.

Understanding the barriers to and enablers of real-world utility and practice is crucial if app developers want pragmatic uptake and efficacy of interventions. Sufficient resourcing may be a factor in longitudinal follow-up; however, to leverage the impact and cost-effectiveness of interventions, studies should factor longer-term assessments in the design process from conceptualization. Nevertheless, easier and low-cost measures of enactment are possible and suggested for future research, including questionnaires on the frequency of using skills taught during the intervention.

### Interpreting Results Using a Logic Model

As part of this review, we developed a logic model to facilitate the process of gathering and integrating studies of complex interventions to better inform our interpretations of cumulative results. The logic model included synthesized data capture and engagement methods used in each study. Theoretically, logic models need moderating or mediating factors to understand how the pathway develops. In these studies, there was a common strength-based approach, such as skill development, confidence, satisfaction, and self-efficacy. Overall, the heterogeneous nature of the data collection meant that we were unable to undertake a meta-analysis; however, the range of methods and types of data collection is useful in guiding future web-based interventions targeting this population group and helping decision-makers understand the rationale for how interventions are expected to work and what enablers keep participants engaged to ultimately achieve the intended outcomes.

There is a need for a greater understanding of the individual-level, real-world factors affecting engagement in home and minimal contact practice interventions to ascertain how participants experience interventions and how this relates to their outcomes [19]. Exit and follow-up interviews can provide a deeper understanding of participants’ experiences to strengthen real-life sustained engagement in that modality. Experience of an intervention needs to be user-friendly, accessible, and positive, which should be considered in promoting material that is most effective and helpful for users to engage from the outset.

### Limitations of This Systematic Review

As the studies in this review were diverse in terms of study design, therapeutic intervention approach and delivery, length of follow-up, and outcome measures, we summarized the engagement data using the CAPE framework but were unable to perform a meta-analysis of the data. Attrition rates were high in many studies, and the number of participants was small, particularly in some pilot studies. We were unable to report this in terms of increasing our understanding of sustained practice as there was limited follow-up in most studies. There are inconsistent reports and terminology regarding engagement behavior. Inconsistencies in language between studies and interchangeability of terms, for example, attrition, withdrawal, dropout, and loss to follow-up, make direct comparison and systematic analysis challenging. Another potential limitation of this review is the lack of inclusion of studies in languages other than English. In addition, the protracted nature of systematic reviews means that the original search was concluded in 2020 and was affected by delays because of the COVID-19 pandemic. Since then, additional studies may have been published and not included in this review but would not necessarily affect our general conclusions or implications for using the logic model or reporting matrix.

### Strengths and Future Work

The ability to leverage several frameworks enhanced this systematic review. The SWiM guidelines, part of the Cochrane methods, directed our synthesis and reporting. In addition, the CAPE framework provided an evidence-based approach to reporting on intervention engagement; using this framework, we were able to propose clear metrics for future reporting. It is recommended that future research provide engagement analytics to more clearly delineate between study and intervention compliance, particularly longer-term enactment or sustained practice to reflect pragmatic efficacy. The research team has a strong focus on research translation; therefore, the incorporation of a logic model provides a clear pathway for decision-makers, such as policy makers and commissioners, to interpret and guide the key constructs and evaluation metrics in future digital interventions in this field of research.

There is substantial evidence that psychological programs delivered on the web can be effective in treating and preventing mental health problems; however, the uptake of these programs can be suboptimal, and there remains a lack of evidence on how to increase engagement with evidence-based programs [45]. Poor adherence is a common feature of web-based mental health programs, which affects intervention outcomes [45] and limits real-world efficacy. Eisenstadt et al [42] discussed in their recent systematic review that adherence and retention continue to be challenges to the quality of research, with little or no information about reasons for dropouts given across studies. Further research is needed to unpack the key constructs of experience, including microlevel reporting and qualitative, phenomenological investigation via one-to-one postprogram interviews. Future reporting of DMHI using the CAPE framework could be used to ascertain the cost-benefit of an intervention; that is, if the conversion, recruitment, retention, and participation rates are high, the intervention is likely to be more cost-effective. However, this must be considered alongside the efficacy of the intervention and real-world application. The motivation for engaging in research studies is very different from real-world engagement experiences.

Advances in technology, particularly the internet, have proven to be an effective tool for building individual skills as it is inexpensive and accessible, both geographically and temporally. Despite promising results, internet interventions are still not widely disseminated or well-integrated into health services; successfully doing so will, in part, depend on engagement. As mental health apps have proliferated, choosing among them has become increasingly challenging for not only patients but also clinicians [46]. To address this, we need to understand the barriers and enablers for the delivery and sustainability of internet interventions in practice [17], as well as how we can engage not only participants but also health practitioners to support and disseminate effective interventions. This increased understanding will enable appropriate investment, optimization, and uptake of targeted well-being programs, such as those developed for perinatal women, with the ultimate aim of preventing poor mental health among women and their children.

### Conclusions

To invest in accessible, long-term, sustainable health solutions, researchers, policy makers, and clinicians must identify optimal interventions that can be targeted to help specific risk groups or in specific contexts. Advances in technology, particularly the internet, have proven to be an effective tool for building individual skills as it is inexpensive and widely accessible. Pregnancy and the postnatal period can be times of increased psychological distress; therefore, it is an optimal time to intervene with strength-based tools to build affirmative self-efficacy. Although several studies in this field demonstrate efficacy, few robustly explore the construct of engagement, and in particular, there is limited evidence of the long-term enactment of the strategies learned. Our results indicate a disparity in the reporting of short- and long-term participant engagement behaviors, and we recommend the adoption of standardized metrics for reporting DMHI engagement in both research and real-world settings. This systematic review provides a framework for understanding the pathways for enhancing the mental well-being of mothers and their infants. With the world experiencing an endemic escalation in poor mental health across the life course, both in low- and high-income countries [55], it is imperative that we create practical, evidence-based, cost-effective, and scalable solutions to protect current and future generations.

## Appendix

Multimedia Appendix 1 Search strategy and study selection.

Multimedia Appendix 2 Detailed study characteristics.

## Abbreviations

CONSORT: Consolidated Standards of Reporting Trials
CAPE: Connect, Attend, Participate, and Enact
DMHI: digital mental health intervention
PRISMA: Preferred Reporting Items for Systematic Reviews and Meta-Analyses
PROSPERO: International Prospective Register of Systematic Reviews
RCT: randomized controlled trial
SWiM: Synthesis Without Meta-analysis

## Data Availability

Data presented in this study are available on reasonable request from the corresponding authors.
