# Supplementary material for: Understanding Engagement in Digital Mental Health and Well-being Programs for Women in the Perinatal Period: Systematic Review Without Meta-analysis
Source: J Med Internet Res. 2022 Aug 9;24(8):e36620. doi: 10.2196/36620 (PMC9399849; doi:10.2196/36620)
Supplement: Multimedia Appendix 1 [file jmir_v24i8e36620_app1.docx]

## Supplementary Information

## Search Strategy and Study Selection

The search was performed by the first author (J.D.), using Medline, Embase, PsycINFO, Scopus, Web of Science, Google Scholar (advanced) and MeSH on Demand for all English language articles published from database from 1^st^ January 2010 through to May 2020.

| **Database** | **OVID MEDLINE** | | | | |
| --- | --- | --- | --- | --- | --- |
| **Date** | 29/05/2020 | | | | |
| **Limits** | English language; last 10 years | | | | |
| **No. Results** | **669** | | | | |
| **#** | **Field** | **Concept** | **Terms** | **Results** |  |
| **1** | Title OR Abstract | Wellbeing | Psycho* OR wellbeing OR well-being OR mental health OR stress OR postnatal depression OR depression OR depress* OR anxiety OR anxi* OR distress | 907156 |  |
| **2** | **Title OR Abstract** | **Digital** | **ehealth OR eHealth OR e-health OR mhealth OR mHealth OR m-health OR web based OR web-based OR technology OR digital technology OR mobile technology OR internet OR online OR on-line OR digi* OR telehealth OR non pharmacological or non-pharmacological OR** computerized cognitive behaviour therapy OR cCBT OR computerised CBT OR electronic mental health OR eMH OR e-MH OR electronic MH | 424142 |  |
| **3** | Title OR Abstract | Programs | program* OR therapy OR treatment OR training OR intervention | 2871360 |  |
| **4** | Title OR Abstract | Perinatal | Preg* OR perinatal OR postnatal OR antenatal | 243024 |  |
| **5** | MeSH Headings | Pregnancy wellbeing programs | wellbeing.mp. or Mental Health/ AND distress or anxi* or depress* AND internet.mp. or Internet-Based Intervention/ or Internet/ and pregnancy or antenatal | **15** |  |
| **6** |  | Wellbeing programs | 1 AND 3 | 332200 |  |
| **7** |  | Digital perinatal wellbeing programs | 6 AND 2 AND 4 | 654 |  |
| **8** |  | Digital perinatal wellbeing programs | 5 AND 7 | **669** |  |

| **Database** | **OVID Embase** | | | | |
| --- | --- | --- | --- | --- | --- |
| **Date** | 22/05/2020 | | | | |
| **Limits** | English language; last 10 years | | | | |
| **No. Results** | **1,279** | | | | |
| **#** | **Field** | **Concept** | **Terms** | **Results** |  |
| **1** | Title OR Abstract | Wellbeing | Psycho* OR wellbeing OR well-being OR mental health OR stress OR postnatal depression OR depression OR depress* OR anxiety OR anxi* OR distress | 1324182 |  |
| **2** | **Title OR Abstract** | **Digital** | **ehealth OR eHealth OR e-health OR mhealth OR mHealth OR m-health OR web based OR web-based OR technology OR digital technology OR mobile technology OR internet OR online OR on-line OR digi* OR telehealth OR non pharmacological or non-pharmacological OR** computerized cognitive behaviour therapy OR cCBT OR computerised CBT OR electronic mental health eMH OR e-MH OR electronic MH | 626088 |  |
| **3** | Title OR Abstract | Programs | program* OR therapy OR treatment OR training OR intervention | 4695409 |  |
| **4** | Title OR Abstract | Perinatal | Preg* OR perinatal OR postnatal OR antenatal | 400568 |  |
| **5** | MeSH Headings | Pregnancy wellbeing programs | wellbeing.mp. or Mental Health/ AND distress or anxi* or depress* AND internet.mp. or Internet-Based Intervention/ or Internet/ and pregnancy or antenatal | **52** |  |
| **6** |  | Wellbeing programs | 1 AND 3 | 542266 |  |
| **7** |  | Digital perinatal wellbeing programs | 6 AND 2 AND 4 | 1227 |  |
| **8** |  | Digital perinatal wellbeing programs | 5 AND 7 | **1279** |  |

| **Database** | **OVID PsychInfo** | | | | |
| --- | --- | --- | --- | --- | --- |
| **Date** | 29/05/2020 | | | | |
| **Limits** | English language; last 10 years | | | | |
| **No. Results** | **271** | | | | |
| **#** | **Field** | **Concept** | **Terms** | **Results** |  |
| **1** | Title OR Abstract | Wellbeing | Psycho* OR wellbeing OR well-being OR mental health OR stress OR postnatal depression OR depression OR depress* OR anxiety OR anxi* OR distress | 528127 |  |
| **2** | **Title OR Abstract** | **Digital** | **ehealth OR eHealth OR e-health OR mhealth OR mHealth OR m-health OR “web based” OR web-based OR technology OR “digital technology” OR “mobile technology” OR internet OR online OR on-line OR digi* OR telehealth OR “non pharmacological” OR non-pharmacological OR “**computerized cognitive behaviour therapy” OR cCBT OR “computerised CBT” OR “electronic mental health” OR eMH OR e-MH OR “electronic MH” | 149128 |  |
| **3** | Title OR Abstract | Programs | program* OR therapy OR treatment OR training OR intervention | 514564 |  |
| **4** | Title OR Abstract | Perinatal | Preg* OR perinatal OR postnatal OR antenatal | 33191 |  |
| **5** | MeSH Headings | Pregnancy wellbeing programs | wellbeing.mp. or Mental Health/ AND distress or anxi* or depress* AND internet.mp. or Internet-Based Intervention/ or Internet/ and pregnancy or antenatal | **13** |  |
| **6** |  | Wellbeing programs | 1 AND 3 | 199131 |  |
| **7** |  | Digital perinatal wellbeing programs | 6 AND 2 AND 4 | 258 |  |
| **8** |  | Digital perinatal wellbeing programs | 7 AND 5 | **271** |  |

| **Database** | **Web of Science** | | | |
| --- | --- | --- | --- | --- |
| **Date** | 22/05/2020 | | | |
| **Limits** | English language; last 10 years; document type (article; proceedings; review; meeting abstract; book chapter; editorial material) | | | |
| **No. Results** | 1,014 | | | |
| **#** | Field | Concept | Terms | Results |
| **1** | **Title OR Abstract**  **Boolean/ Phrase** | **Wellbeing** | **wellbeing  OR  well-being  OR  mental  AND health  OR  wellness OR** stress OR postnatal depression OR depression OR anxiety OR depress* OR anxiety OR anxi* OR distress | 1,651,187 |
| **2** | Title OR Abstract  Boolean/ Phrase | Digital | **ehealth OR eHealth OR e-health OR mhealth OR mHealth OR m-health OR “web based” OR web-based OR technology OR “digital technology” OR “mobile technology” OR internet OR online OR on-line OR digi* OR telehealth OR “non pharmacological” OR non-pharmacological OR “**computerized cognitive behaviour therapy” OR cCBT OR “computerised CBT” OR “electronic mental health” OR eMH OR e-MH OR “electronic MH” | 1,818,494 |
| **3** | Title OR Abstract  Boolean/ Phrase | Programs | programs  OR  interventions  OR  treatment  OR  therapy | 4,391,204 |
| **4** | Title OR Abstract  Boolean/ Phrase | Perinatal | perinatal  OR  antenatal  OR  postnatal  OR  pregnancy | 265,431 |
| **6** |  | Wellbeing programs | 1 AND 3 | 502,799 |
| **7** |  | Digital wellbeing programs | 6 AND 2 | 31,744 |
| **8** |  | Digital perinatal wellbeing programs | 7 AND 4 | 1,014 |

| **Database** | **Scopus** | | | |
| --- | --- | --- | --- | --- |
| **Date** | 29/05/2020 | | | |
| **Limits** | English language; last 10 years; document type (article; review; conference paper; book chapter; editorial material) | | | |
| **No. Results** | 277 | | | |
| **#** | Field | Concept | Terms | Results |
| **1** | **Title OR Abstract**  **Boolean/ Phrase** | **Wellbeing** | **wellbeing  OR  well-being  OR  mental  AND health  OR  wellness OR** stress OR postnatal depression OR depression OR anxiety OR depress* OR anxiety OR anxi* OR distress | **92,805** |
| **2** | Title OR Abstract  Boolean/ Phrase | Digital | **ehealth OR eHealth OR e-health OR mhealth OR mHealth OR m-health OR “web based” OR web-based OR technology OR “digital technology” OR “mobile technology” OR internet OR online OR on-line OR digi* OR telehealth OR “non pharmacological” OR non-pharmacological OR “**computerized cognitive behaviour therapy” OR cCBT OR “computerised CBT” OR “electronic mental health” OR eMH OR e-MH OR “electronic MH” | **2,717,556** |
| **3** | Title OR Abstract  Boolean/ Phrase | Programs | programs  OR  interventions  OR  treatment  OR  therapy | **5,026,183** |
| **4** | Title OR Abstract  Boolean/ Phrase | Perinatal | perinatal  OR  antenatal  OR  postnatal  OR  pregnancy | **346,060** |
| **7** |  | Wellbeing programs | 1 AND 3 | **53,397** |
| **8** |  | Digital wellbeing programs | 7 AND 2 | **4,980** |
| **9** |  | Digital perinatal wellbeing programs | 8 AND 4 | 277 |

Search results in EndNote = 3,473

After de-duplication = **2,795**

| **Database** | **Google Scholar: Advanced** | | | |
| --- | --- | --- | --- | --- |
| **Date** | 22/05/2020 | | | |
| **Limits** | last 10 years | | | |
| **No. Results** | 3,330 | | | |
| **#** | Field | Concept | Terms | Results |
| **1** |  | Digital perinatal wellbeing programs | Digital perinatal wellbeing programs | 3,330 |

| **Database** | **MeSH on Demand** | | | |
| --- | --- | --- | --- | --- |
| **Date** | 22/05/2020 | | | |
| **Limits** | last 10 years | | | |
| **No. Results** |  | | | |
| **#** | Field | Concept | Terms | Results |
| **1** |  | Digital perinatal wellbeing programs | Digital perinatal mental health programs | 10 |
