# Supplementary material for: Understanding Engagement in Digital Mental Health and Well-being Programs for Women in the Perinatal Period: Systematic Review Without Meta-analysis
Source: J Med Internet Res. 2022 Aug 9;24(8):e36620. doi: 10.2196/36620 (PMC9399849; doi:10.2196/36620)
Supplement: Multimedia Appendix 2 [file jmir_v24i8e36620_app2.docx]

# **Understanding engagement in digital mental health and wellbeing programs for women in the perinatal period: A Systematic Review without Meta-Analysis (SWiM)**

| ***Table S1:****Group 1: Randomised Controlled Trials (n.6)* | | | | | | | | |
| --- | --- | --- | --- | --- | --- | --- | --- | --- |
| **Study**  **(Country)** | **Intervention type, format & duration** | **Study aims**  **Sample size** | **Therapeutic Approach** | **Mental Health Outcomes Measures** | **Mental Health Outcomes / Results** | **Limitations** | **Strengths** | **Risk of Bias**** |
| Ashford, M. T. [13]  2018  Australia / United Kingdom | Self-guided  Internet-based *What Am I Worried About* (iWaWa)  9 modules | Assess the feasibility and acceptability of iWaWA among postpartum women with anxiety  89 participants | CBT and mindfulness practices  Plus optional weekly telephone support | Anxiety symptoms assessed at baseline, 8 weeks and 12 weeks post randomisation | Treatment acceptability  Client Satisfaction questionnaire  No significant difference between treatment and control groups in GAD scores | Only 2 women completed all 9 modules  Convenience sampling  Homogenous sample | Constructive investigation of format improvements i.e., too long, needs app format  Drop outs invited for interview | Low |
| Chan, K. L. [50]  2019  Hong Kong | Smartphone-based mobile app | Assess the difference in the levels of antenatal and postnatal depression in participants  660 participants | Psychoeducational mobile app  Replicated antenatal classses | Edinburgh Postnatal Depression Scale (EPDS)  Baseline sureys: anxiety and stress levels, health-related quality of life (QoL), and demographic characteristics | Associations were  found between participation in the intervention and reduced depression | Short postpartum period  First time mothers not all mothers | Potentially a cost-effective alternative | Low |
| Gammer, I. [52]  2020  United Kingdom | Online compassion-based intervention  *Kindness for Mums Online (KFMO)*  5-6 weeks | Assess the effect of the intervention on participants’ wellbeing  206 participants | Compassion-based intervention | Wellbeing: Warwick–  Edinburgh Mental Well-being Scale (WEMWBS)  SCS-SF  The Forms of Self-  Criticizing/Attacking and Self-Reassuring Scale (FSCRS)  DASS-21 | Effect of the intervention on well-being was small and robustly increased self-compassion relative to control | Relatively high attrition rates and limited generalizability to more diverse samples – imputed data for missing variables.  Pragmatic trial. | Reporting of attrition  Researchers blinded to RCT arms | Low |
| Guo, L. [53]  2020  China | A Chinese version of the MBSP program  10 hours of training with 36 episodes  6-week internet-based | Assess the effect of the mindful self-compassion intervention on preventing postpartum depression  in a group of symptomatic pregnant women  314 participants | Mindful Self-Compassion Program | EPDS, the State-Trait Anxiety Inventory I and II, and Beck  Depression Inventory II (BDI)  MAAS; SCS; Wellbeing Index (WHO-5); Chinese Parenting Stress Index; Comprehensive Parenting Behavior Questionnaire 1-year Chinese version; Infant Behavior Questionnaire | The intervention group showed significant improvement in depressive  and anxiety behaviors  The intervention group became more  mindful and self-compassionate at 3 months and 1 year postpartum | When interpreting the infant's temperaments, changes in the  mother's perception should be considered. When mothers are more attentive  and positive toward their infants, it is more likely for them to bias the  infant's positive activities. | The dropout rate was low,  and the participants who completed this program rated it as highly  useful. This program is feasibleand acceptable for women experiencing stress during pregnancy  and motherhood. | Low |
| Krusche, A. [54]  2018  United Kingdom | Condensed online version of an eight-week mindfulness course.  *‘Be Mindful Online’* general mindfulness  4-week online | Evaluate the potential of an online mindfulness course for expectant participant mothers  185 participants | Formal and informal meditation practices such as body scan, mindful movement, breathing space and mindful eating, through videos and assignments. | Primary outcome was change in perceived stress from pre- to post intervention. Secondary outcomes were changes in general mood (anxiety and depression), pregnancy-related distress and labour worry.  PSS; GAD-7; EPDS; *Tilburg Pregnancy Distress Scale* (TPDS; *Oxford Worries about Labour Scale* (OWLS); Mindfulness practice | Participants had a significant improvement in general stress compared with their waitlist peers.  Course completers reported greater improvements in mood, post-intervention | Levels of drop out from the course were very high.  Suggests that relatively few women allocated to receive a generic online mindfulness course during pregnancy find it sufficiently engaging to continue to participate in online sessions | Feasibility issues identified – course needs modification to prevent high drop outs.  Comparison between completers and non-completers.  Important to explore the reasons that people do not complete the online mindfulness course | Low |
| Shorey, S. [51]  2017  Singapore | Mobile app for psychoeducation and postnatal depression  *'Home-but not Alone'* | To examine the effectiveness of the program to improve participant parenting outcomes    250 participants (couples) | Parental self-efficacy primary outcome | Parental self-efficacy, social support, postnatal depression and parenting satisfaction  PSE Parenting Efficacy Scale; Perceived Social Support for Parenting scale: Edinburgh PND Scale; What Being the Parent of a New Baby is Like scale | The intervention group had statistically significant improvements for parental self-efficacy, social support and parenting satisfaction at 4 weeks postpartum. No effect on depression. | The majority of participants were educated, middle-income.  Minimal follow-up. | Program could be supported by nurses as routine care.  Fathers included.  Fostered a virtual community for parents | Low |

| ***Table S2:*** *Group 2: Non RCTs – case series, open trial, quasi experimental (n.3)* | | | | | | | | |
| --- | --- | --- | --- | --- | --- | --- | --- | --- |
| **Study**  **(Country)** | **Intervention type, format & duration** | **Study aims**  **Sample size** | **Therapeutic approach eg CBT** | **Mental Health Outcomes Measures** | **Mental Health Outcomes / Results** | **Limitations** | **Strengths** | **Risk of Bias**** |
| Corno, G. [32]  2018  Spain | 5-week online self-applied positive psychology intervention specifically adapted for pregnant women  4 modules | Examine the effect of a positive psychology web-based intervention on indices of participants’ prenatal wellbeing  6 participants  *Case series design* | 4Each module includes a brief psychoeducation unit (i.e., mindfulness and self-acceptance; savoring; connectedness and social support; optimism and life satisfaction) and a positive psychology exercise. | Measures of women’s mental well-being, depression, pregnancy-related anxiety, life satisfaction, and social support. Single-item related well-being measures assessed weekly  WEMWBS; PHQ-9; PRAS: MSPSS; | Potential effects of the intervention on supporting mental well-being and decreasing depressive symptomatology | Limited sample size  Only 5 weeks plus 1 timepoint post-itnervention  No control | Positive preliminary results | Low |
| Felder, J. N. [33]  2017  United States | Internet program plus weekly phone coaching sessions, individually or group  *Mindful Mood Balance [MMB]* program  8 weeks | Examine the feasibility, acceptability, and  preliminary outcomes of MMB for use with pregnant women at risk for depressive relapse  37 participants  *Open trial – no control group* | Mindfulness-based cognitive therapy (MBCT)  includes mindfulness, yoga and CBT | Predicted that women would not demonstrate significant worsening  of depression symptom severity during MMB.  Structured Clinical Interview for DSM-IV Axis I Disorders  Psychiatric Diagnostic  Screening Questionnaire (PDSQ  Patient Health Questionnaire (PHQ-9)  score ≤ 12. | Sustained minimal to  mild depressive symptom severity over the course of the program | No control group  No follow up after post-test ie depressive relapse  12 participants lost to follow up  4 discontinued  Completion rate 57%  Time constraints main reason for withdrawing  Low uptake of phone coacing | Comprehensice reporting of ‘engagement’ but not sustained engagement | Uncertain |
| Tsai, Y. J. [34]  2018  Taiwan | Web-based modules:  1) web-based maternity health records,  2) antenatal health education,  3) self-management journals, and  4) infant birth records. | Investigate the effectiveness of a web-based antenatal  care and education system on pregnancy-related stress, general self-efficacy, and satisfaction with antenatal care  135 pregnant women  *Quasi-experimental design* | Antenatal care and education system on pregnancy-related stress, general self-efficacy | Pregnancy stress and general self-efficacy  General Self-Efficacy scale (GSE)  Pregnancy Stress (PSRS-36)  Satisfaction with healthcare | Intervention group: significantly lower pregnancy-related stress  and significantly higher self-efficacy.  Improved women’s satisfaction with antenatal care. | Quasi-experimental  design was used; thus, the participants were not  randomly assigned. Limited  generalizability for women with high-risk pregnancies.  No post-birth follow up. | Reporting of potential biases in study design. Tailored information to meet the women’s needs according to their  different gestational ages. | Low |

| ***Table S3:*** *Group 3: Pilot studies (n.7)* | | | | | | | | |
| --- | --- | --- | --- | --- | --- | --- | --- | --- |
| **Study**  **(Country)** | **Intervention type, format & duration** | **Study aims**  **Sample size** | **Therapeutic approach eg CBT** | **Mental Health Outcomes Measures** | **Mental Health Outcomes / Results** | **Limitations** | **Strengths** | **Risk of Bias**** |
| Ayers, S. [35]  2015  United Kingdom | Brief on-line self-help intervention – 5 components considered effective in challenging negative beliefs | Assess positive mood in participating mothers of babies and toddlers  80 participants | CBT questions | Anxiety and depression (HADS); Mood checklist (Uni of Wales Inst of Sci & Tech Mood adjective Checklist); Self-esteem Scale | Improved mood in both groups post-intervention | Brief – only one session  No long-term follow up  Acceptabliity question open-ended | Pilot study – recommendations for larger, longitudinal study | Uncertain |
| Barrera, A. Z. [36]  2015  United States | Automated self-help Internet intervention  8 lessons – accessible anytime | Assess efficacy of the intervention to reduce the risk of postnatal depression in participating women.  111 participants | Mood management internet intervention – CBT framework adapted from F2F intervention | Center for Epidemiologic Studies-Depression Scale  EPDS  Major Depressive Episode Screener | The effect of the prevention intervention condition failed to reach significancel. However, the observed coefficient trended in the hypothesized direction | High attriction and low engagement. Large number recruited but failed to log in – discussed. | Women from 23 countries – diverse  Reported missing data and those that did not log in | Low |
| Barrera, A. Z. [37]  2019  United States | Minimal contact automated text messaging  *BabyText* program | Assess acceptability of a text messaging program to prevent postpartum depression  10 participants (pregnant and postpartum women) | SMS tips based on CBT framework | Mood rating Pateint Health Questionnaire (PHQ-4) – measures depression and anxiety (n 7) | Need to personalise the tips – personalisation important to users | Cross-sectional not longitudinal  Small sample size – only 7 women provided post-int feedback  Limitation (low feedback response rate) | Ethnically/racially diverse group | Uncertain |
| Cornsweet B.C. [38]  2013  New Zealand | Intervention – self guided  15 steps, each of which takes 45 mins | Assess feasibility and acceptability:  Study 1 – (n =6) effects of a single teaching and biofeedback session on maternal and fetal biofeedback  Study 2 – (n =9) effect of consumer satisfaction | Computerised self-help package teaching relaxation and mindfulness skills, using biofeedback mindfulness | Study 1- Fetal heart rate monitoring & mat physiological data (eg respiration rate)  Study 2 – PSS10, EPDS, STAI (Stait-Trait Anxiety), CSES (Coping Self-Efficacy), MAAS | Study 1 – 1 session of instruction and biofeedback appeared to produce detectable physiological reponse  Study 2 – positive response and positive changes | Small sample sizes, no follow up.  No control – pre and post tests  Statistical analyses limited – sample size  Limited recommended follow up i.e., full trial | Includes physiological changes and biofeedback  Women found the program enjoyable  Feasibility study only | Uncertain |
| Duffecy, J. [39]  2019  United States | 8-week online prevention intervention  Website plus initial phone call  16 core didactic lessons plus 3 postpartum booster sessions and 5 associated tools | Assess a CBT peer support intervention to prevent postnatal depression in participants  24 participants | CBT | Hamilton Depression Rating Scale (HDRS); Inventory of Depression and Anxiety Symptoms (IDAS); Patient Health Questionnaire (PHQ-8) | At 6 weeks postpartum, only 4% (1/24) met the criteria for PPD. | Small sample size  Short follow up duration | Assessors were blinded to site arm  and intervention usage.  Includes adherence data table | Low |
| Fonseca, A. [40],  2019  Portugal | Self-guided, web-based intervention to prevent postpartum depression symptoms  *Be a Mom*  5 modules | Explore the processes underlying therapeutic change for participants in the intervention  194 participants | CBT: acceptance  and compassion-based approaches (third-wave approaches) applied to the perinatal  context. | Measures of depressive symptoms, emotion regulation abilities, psychological flexibility and self-compassion  EPDS  DERS-SF  Acceptance and Action Questionnaire-II  SCS-SF | A significantly greater decrease in the levels of emotion regulation  difficulties and a significant greater increase in the levels of self-compassion compared to the control group. | Challenges with randomisation differences in groups  Assessment times limited | Providing some insights into the processes that underlie treatment response to *Be a Mom*, this study highlights the role of the targeted third-wave  processes applied to the perinatal context | Secondary analysis of the results of a pilot randomized trial conducted to evaluate Be a Mom’s feasibility and acceptability.  Uncertain |
| Matvienko-Sikar, K. [41]  2017  Ireland | Online mindfulness and gratitude intervention 4 times a week for 3 weeks | Assess the effect of a novel gratitude and mindfulness-based intervention on prenatal stress, cortisol levels, and wellbeing in participating women.  46 pregnant women | Online gratitude and mindfulness based intervention on prenatal stress, cortisol levels, and well-being | Prenatal Distress Scale (PDQ5)  Mindfulness Attention Awareness Scale (MAAS) Satisfaction with Life Scale (SWLS)  Salivary cortisol samples at 3 sampling periods during the study: baseline (Time 1), 1.5 weeks later (Time 2) and 3 weeks later (Time 3). | Intervention participants demonstrated significant reductions in prenatal stress in comparison to the control condition  Effects on a biomarker of stress, cortisol, were also observed | Low levels of participant recruitment  Small sample size.  The majority of participants were highly educated, engaged in positive health behaviours, had planned, low-risk pregnancies and were in private antenatal care; all participants were also in a relationship.  Inaccurate saliva sampling | Inclusion of stress biomarker.  Brief at-home intervention | Low |
